# β‐Mercaptoethanol‐Enabled Long‐Term Stability and Work Function Tuning of MXene

**DOI:** 10.1002/smsc.202200057

**Published:** 2022-10-02

**Authors:** Hongyue Jing, Benzheng Lyu, Yingqi Tang, Sungpyo Baek, Jin-Hong Park, Byoung Hun Lee, Jin Yong Lee, Sungjoo Lee

**Affiliations:** ^1^ SKKU Advanced Institute of Nanotechnology (SAINT) Sungkyunkwan University Suwon 440-746 Korea; ^2^ Department of Electrical and Electronic Engineering The University of Hong Kong Hong Kong 518057 China; ^3^ Department of Chemistry Sungkyunkwan University Suwon 16419 Korea; ^4^ Department of Electrical Engineering Pohang University of Science and Technology Pohang 37673 Korea; ^5^ Department of Nano Engineering Sungkyunkwan University Suwon 440-746 Korea

**Keywords:** MXene, stability, Ti—S bond, work function, β-mercaptoethanol

## Abstract

The oxidation degradation by unsaturated metal atoms or dangling bonds at MXene edges and defects severely hinders the practical application of MXene. Herein, a passivation scheme for Ti_3_C_2_T_*x*_ MXene is demonstrated by utilizing a sulfhydryl‐containing molecule, β‐mercaptoethanol (BME), which can significantly suppress the Ti_3_C_2_T_*x*_ oxidation in various environments, including long‐term storage of Ti_3_C_2_T_*x*_ aqueous dispersions (2 m), single‐layer Ti_3_C_2_T_*x*_‐based devices in humid air (2 m), and high‐temperature environment (12 h). Notably, the nonionic BME does not cause aggregation but maintains the 2D morphology of Ti_3_C_2_T_*x*_. A comprehensive investigation of the protection mechanism through density functional theory (DFT) calculations and experimental characterizations reveals that BME is adsorbed especially at the edges and surface defects of MXene (binding energy −1.70 and −1.05 eV), where the degradation starts. Further, the electron‐donating effect of sulfhydryl groups tunes the work function of Ti_3_C_2_T_*x*_ from 4.70 to 4.39 eV, resulting in improved carrier‐transport performances in MoS_2_ field‐effect transistors owing to band alignment, where BME–Ti_3_C_2_T_*x*_ serves as the source electrode. The described methodology can largely contribute to the ultralong service life of 2D Ti_3_C_2_T_*x*_ without affecting its excellent properties, thereby promoting the practical application of this emerging material.

## Graphical abstract

A β‐mercaptoethanol–MXene (BME–Ti_3_C_2_T_*x*_) composite with long‐term stability, 2D features, and excellent electrical properties, enabled by the original strong interaction between sulfhydryl (—SH) and MXene, is demonstrated. BME–Ti_3_C_2_T_*x*_ exhibits a lower work function than that of pristine Ti_3_C_2_T_*x*_ and is a better source electrode material in n‐type MoS_2_ field‐effect transistors.

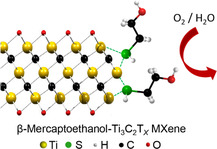

## 1 Introduction

MXene, an emerging family of 2D‐layered transition‐metal carbides/nitrides with a general chemical formula of M_*n*+1_X_*n*_T_*x*_ (*n* = 1–4), has exhibited numerous attractive physical and chemical properties such as metallic conductivity,^[^ ^1^ ^]^ water dispersibility,^[^ ^2^ ^]^ high optical transparency,^[^ ^3^ ^]^ good mechanical properties,^[^ ^4^ ^]^ modifiable surface functional groups,^[^ ^5^ ^]^ and tunable work function.^[^ ^6^ ^]^ Since the discovery of MXene in 2011, the MXene family has exhibited superior performances in various applications such as energy storage,^[^ ^7^ ^]^ electronics,^[^ ^8^ ^]^ photothermal conversion,^[^ ^9^ ^]^ electromagnetic interference shielding,^[^ ^10^ ^]^ sensors,^[^ ^11^ ^]^ water purification,^[^ ^12^ ^]^ and desalination.^[^ ^13^ ^]^ In addition to the superior performances, considering the abundant functional groups on the surface, MXene can be synthesized by a solution method and processed in the aqueous phase, which could provide low‐cost and large‐scale MXene applications.^[^ ^14^ ^]^ The surface functional groups also stabilize the active early transition‐metal atoms on the surface of MXene, such as Ti, V, Cr, and Nb.^[^ ^15^ ^]^ However, the MXene flakes synthesized by the solution method must have edges and numerous defects, where the metal atoms are unsaturated, referred to as dangling bonds; thus, MXene can be easily oxidized by oxidants such as H_2_O and O_2_ (Figure S1a,b, Supporting Information).^[^ ^16^ ^]^ Through this spontaneous oxidation reaction, MXene gradually degrades to metal oxides and amorphous carbon in humid air or water dispersion, which can severely affect its stability during storage and processing and long‐term effectiveness in practical applications.^[^ ^17^ ^]^

Previous studies demonstrated effective strategies to delay the oxidation reaction of MXene, which can be roughly summarized into two categories: control of external factors that induce the oxidation reaction and stabilization of MXene by passivating the unsaturated Ti atoms at the defects and edges. For example, the oxidant of MXene, dissolved O_2_ in water, can be removed by degassing using inert gases (Ar or N_2_),^[^ ^18^ ^]^ while another oxidant, H_2_O, can be replaced by dispersing MXene in organic solvents.^[^ ^19^ ^]^ However, the dispersions of MXene in organic solvents without additional additives generally lead to issues of low concentration and low dispersion stability.^[^ ^20^ ^]^ Another factor of the oxidation reaction is the reaction rate. A low temperature (as low as −80 °C)^[^ ^21^ ^]^ slows the oxidation reaction kinetics but has the disadvantages of high energy consumption and low efficiency. Overall, the control of external oxidation factors imposes harsh requirements on the application environment of MXene. In contrast, the method of stabilizing unsaturated Ti atoms has better adaptability because it improves the oxidation resistance of MXene. Mathis et al.^[^ ^22^ ^]^ effectively reduced the defects in the Ti_3_AlC_2_ MAX phase precursor by adding excessive Al during synthesis, thereby largely eliminating the unsaturated Ti atoms in the surface defective part of the MXene sheet, although the edge Ti is still unsaturated. Natu et al.^[^ ^23^ ^]^ raised polyphosphates by capping the edges of MXene to mitigate oxidation. Polyphosphate protection caused MXene aggregation, as high concentrations of salt destroyed the hydration layer of MXene. Zhao et al.^[^ ^24^ ^]^ introduced a sodium L‐ascorbate additive to mitigate the oxidation while preserving the 2D dispersion state of MXene, but the susceptibility to mold limited its application in an unsterilized environment. Therefore, the development of a low‐energy‐consumption method and material that do not require additional repeatable washing processes, especially to simultaneously provide protection in both the storage of dispersion and the application of solid‐state MXene, is still crucial for both fundamental research and practical applications of MXene.

In this study, we demonstrate that β‐mercaptoethanol (BME) can significantly inhibit the degradation of Ti_3_C_2_T_*x*_ MXene, in a colloidal dispersion, under humid air conditions, or even in a harsh 100 °C water. Ti_3_C_2_T_*x*_ was studied because it is the most representative and widely studied MXene, which can be attributed to the highest conductivity in the MXene family, rich surface groups for redox reactions and dispersibility in water, well‐established methods for synthesis and material handling, etc.^[^ ^25^ ^]^ BME was selected because the sulfhydryl groups (—SH) have strong electron‐donating effects and are expected to have strong interactions with the partially positively charged unsaturated Ti atoms at the edges and defects of MXene. This is based on the classic strong interaction between —SH and gold,^[^ ^26^ ^]^ thereby eliminating active sites in the oxidation reaction. —SH has a reducing ability to eliminate oxygen free radicals in water.^[^ ^27^ ^]^ Moreover, compared with the reported ionic protective materials, the nonionic BME seldom destroys the hydration layer of MXene, while the hydrophilic hydroxyl tail is conducive to the good water dispersibility of BME–Ti_3_C_2_T_*x*_ MXene. Thus, 2D BME–Ti_3_C_2_T_*x*_ flakes can be easily obtained by spin‐coating without any further treatments. The oxidation‐suppressing effect of BME and the related mechanism was confirmed by various characterizations such as ultraviolet–visible–near‐infrared (UV–vis–NIR) absorption spectroscopy, atomic force microscopy (AFM), X‐ray photoelectron spectroscopy (XPS), transmission electron microscopy (TEM), Raman spectroscopy, thermogravimetric analysis (TGA) and differential scanning calorimetry (DSC), electron paramagnetic resonance (EPR), and X‐ray diffraction (XRD). Using density functional theory (DFT) calculations and experimental characterizations, we propose that —SH preferentially forms strong bonds (binding energy up to −1.70 eV) with unsaturated Ti atoms at the edges rather than at the surface of MXene, thereby suppressing the oxidation reaction. Furthermore, the electrical properties of BME–Ti_3_C_2_T_*x*_ were investigated by fabricating MXene‐based field‐effect transistors (FETs). BME–Ti_3_C_2_T_*x*_ exhibited better conductivity and considerably better humid air stability than those of pristine Ti_3_C_2_T_*x*_. Finally, the work function of Ti_3_C_2_T_*x*_ was successfully tuned from 4.70 to 4.39 eV by the BME treatment because of the electron‐donating effect of —SH. BME–Ti_3_C_2_T_*x*_ served as a considerably better source electrode material than pristine Ti_3_C_2_T_*x*_ in MoS_2_ FETs owing to the band alignment effect. The described methodology can largely contribute to the long‐term storage of 2D MXene with a low energy consumption, which can likely improve the electronic properties of MXene, thereby promoting the practical application of MXene. In addition, the elucidation of the strong interaction of —SH with MXene and improved stability of MXene at high temperatures is expected to inspire related studies and further expand the research field of MXene.

## 2 Results and Discussion

**Figure 1:**
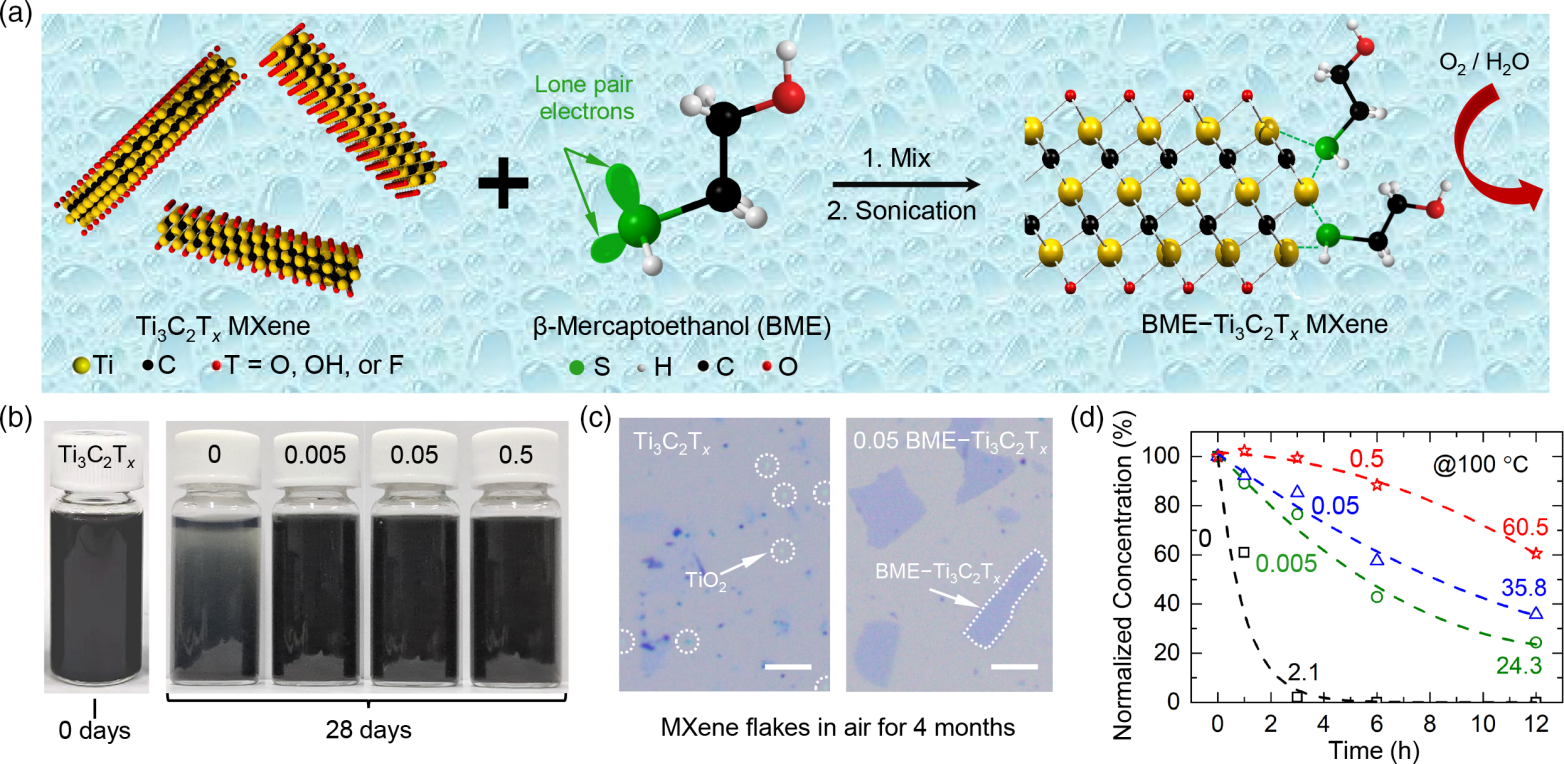
a) Schematic of dispersed 2D Ti_3_C_2_T_*x*_ nanosheets, molecular structure of β‐mercaptoethanol (BME), and BME–Ti_3_C_2_T_*x*_ composite structure to prevent Ti_3_C_2_T_*x*_ attack by O_2_ or H_2_O. b) Digital photographs of the freshly synthesized Ti_3_C_2_T_*x*_ and 0, 0.005, 0.05, and 0.5 BME–Ti_3_C_2_T_*x*_ samples after 28 d of storage at room temperature. c) Optical images of the Ti_3_C_2_T_*x*_ and 0.05 BME–Ti_3_C_2_T_*x*_ flakes on substrates in humid air after 4 m. d) Relationship between the normalized concentration of the 0, 0.005, 0.05, and 0.5 BME–Ti_3_C_2_T_*x*_ aqueous solutions and aging time at 100 °C. Scale bars: 5 μm.

**Figure** **1**a illustrates the aqueous dispersed 2D Ti_3_C_2_T_*x*_ nanosheets, the molecular structure of BME with highlighted lone pairs of electrons, and BME–Ti_3_C_2_T_*x*_ composite structure to prevent MXene attack by O_2_ or H_2_O. Aqueous Ti_3_C_2_T_*x*_ colloids were produced by etching the Al layer from Ti_3_AlC_2_ using HCl/LiF prior to removing extra acid and salts via centrifugation, as illustrated in Figure S2, Supporting Information (see Experimental Section).^[14a]^ Regarding the mechanism of interaction between Ti_3_C_2_T_*x*_ and BME molecules, the S atoms of BME with lone pairs of electrons are considered to preferentially bind with the unsaturated Ti of MXene. The excess BME can also form strong bonds with the defective and perfect MXene surface, which is discussed in detail later. To suppress the oxidation reaction of Ti_3_C_2_T_*x*_, the as‐synthesized Ti_3_C_2_T_*x*_ colloid was separated and diluted with three aliquots of an aqueous BME solution (volume ratio of 1:3), which yielded final BME concentrations in the 10 mL Ti_3_C_2_T_*x*_ colloid of 0.005, 0.05, and 0.5 mol L^−1^. An additional Ti_3_C_2_T_*x*_ suspension diluted by deionized water without BME was regarded as a control. Figure 1b shows images of the as‐prepared pristine Ti_3_C_2_T_*x*_ colloid (0 d) and four Ti_3_C_2_T_*x*_ dispersions containing different amounts of BME after storage at room temperature (25 °C) for 28 d. The vials were shaken by hand and stood for around half an hour before taking photographs. Without BME, the Ti_3_C_2_T_*x*_ dispersion aged for 28 d was almost transparent, which indicates that the concentration was significantly reduced. This reveals the natural oxidation of MXene at room temperature. With the protection of BME, even with the minimum BME amount of 0.005 m, the dispersion exhibited a pitch‐black color, similar to the original Ti_3_C_2_T_*x*_. The concentration of Ti_3_C_2_T_*x*_ in these dispersions was monitored using UV–vis–NIR absorption spectra, as shown in Figure S3, Supporting Information. The absorbances of the Ti_3_C_2_T_*x*_ dispersions with 0.005 (0.005 BME–Ti_3_C_2_T_*x*_), 0.05 (0.05 BME–Ti_3_C_2_T_*x*_), and 0.5 M (0.5 BME–Ti_3_C_2_T_*x*_) of BME were ≈1.92, 2.33, and 2.58 times that of Ti_3_C_2_T_*x*_ in pure water, respectively. Only a slight concentration attenuation and peak red shift were found in aged 0.5 BME–Ti_3_C_2_T_*x*_ compared to that in freshly synthesized pristine Ti_3_C_2_T_*x*_, which demonstrates that BME can effectively restrain the oxidation of Ti_3_C_2_T_*x*_. Figure 1c shows optical microscope (OM) images of a spin‐coated Ti_3_C_2_T_*x*_ (left panel) and 0.05 BME–Ti_3_C_2_T_*x*_ (right panel) on Si/SiO_2_ substrates after storage in a humid air atmosphere (25 °C, relative humidity = 50%) for 4 m. Without BME, Ti_3_C_2_T_*x*_ sheets were oxidized and finally disintegrated in humid air, leaving shiny TiO_2_ particles under OM light. By contrast, the spin‐coated BME–Ti_3_C_2_T_*x*_ maintained its 2D structure after aging, and AFM microscopy further proved that BME–Ti_3_C_2_T_*x*_ retained its original morphology after 4 m (Figure S4, Supporting Information). This reflects the long‐term stability of BME–Ti_3_C_2_T_*x*_ in humid air, and BME did not affect the dispersion of 2D Ti_3_C_2_T_*x*_. Future MXene chemistry may involve high temperatures. Thus, we further analyzed the stabilities of Ti_3_C_2_T_*x*_ and BME–Ti_3_C_2_T_*x*_ in H_2_O at 100 °C. Figure 1d presents the relationship between the concentration of residual MXene and aging time, and the plots were well fitted by single‐exponential decay (the dash lines). Based on the Lambert–Beer law, the concentration of residual MXene was estimated by the maximum absorption peak at ≈750 nm in the UV–vis–NIR spectrum regardless of the gradual change in the extinction coefficient (Figure S5, Supporting Information). Without BME, Ti_3_C_2_T_*x*_ degraded extremely fast at the beginning, then tended to slow down as the oxide layer formed, and was almost completely oxidized in 100 °C H_2_O within 3 h. Under the protection of BME, 24.3%, 35.8%, and 60.5% of Ti_3_C_2_T_*x*_ survived in the 0.005, 0.05, and 0.5 m BME solutions after 12 h, respectively. Without or with a low amount of BME, the oxidation rate of MXene slows down with time, which is due to the gradual thickening of the oxidation layer hindering the oxidation reaction. While the almost linear degradation kinetics of 0.05 BME–Ti_3_C_2_T_*x*_ and the slow initial oxidation rate of 0.5 BME–Ti_3_C_2_T_*x*_ suggest that BME participates in the oxidation reaction and is likely to be oxidized preferentially to Ti_3_C_2_T_*x*_. Additionally, the 0.5 BME–Ti_3_C_2_T_*x*_ after 12 h reaction at high temperature was further stored at room temperature for 14 d, and its UV–vis–NIR spectrum showed only negligible changes (Figure S6, Supporting Information), revealing that BME can still protect Ti_3_C_2_T_*x*_ after high temperature. Therefore, BME is expected to provide high‐temperature chemical reactions and high‐temperature applications of MXene.

**Figure 2:**
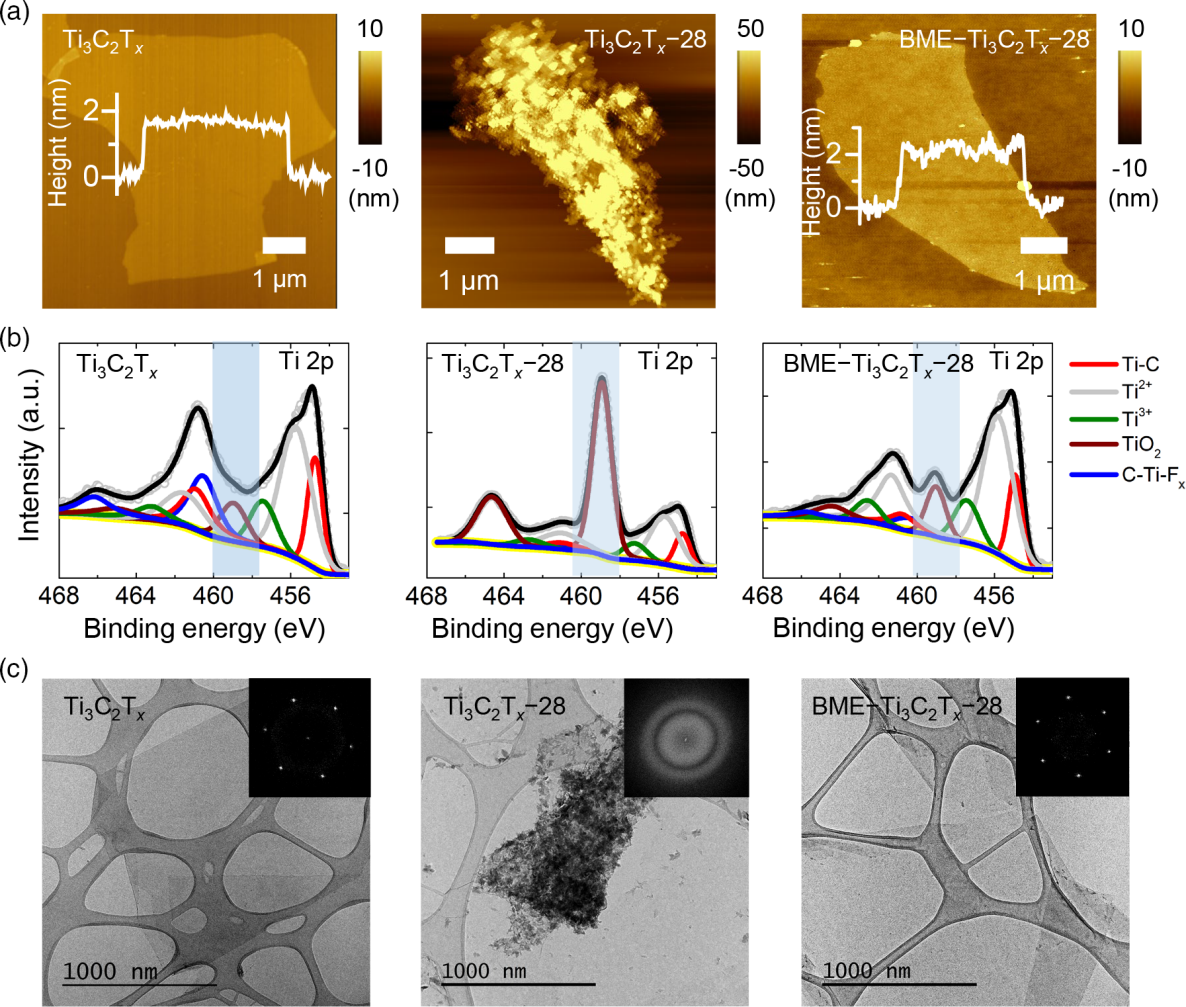
a) Atomic force microscopy (AFM) images, b) Ti 2p X‐ray photoelectron spectroscopy (XPS) spectra, and c) typical transmission electron microscopy (TEM) and fast Fourier transform images (inset) of pristine Ti_3_C_2_T_*x*_ (left), Ti_3_C_2_T_*x*_–28 (middle), and BME–Ti_3_C_2_T_*x*_–28 (right).

Further characterizations were performed to investigate the antioxidation effect of BME using the aforementioned Ti_3_C_2_T_*x*_ and 0.05 BME–Ti_3_C_2_T_*x*_ samples. AFM images of pristine Ti_3_C_2_T_*x*_, Ti_3_C_2_T_*x*_–28 (Ti_3_C_2_T_*x*_ in water after 28 d), and BME–Ti_3_C_2_T_*x*_–28 (Ti_3_C_2_T_*x*_ in the 0.05 m BME solution after 28 d) are presented in **Figure** **2**a from left to right, respectively, with relevant height profiles. The 2D nature of the Ti_3_C_2_T_*x*_ monolayer was confirmed by the AFM image with a height of 1.65 nm.^[^ ^28^ ^]^ While the Ti_3_C_2_T_*x*_–28 lost the 2D feature, exhibiting many particles with sizes of dozens of nanometers. With the protection of BME, even after storage at room temperature for 28 d, the 2D feature of the Ti_3_C_2_T_*x*_ nanosheet was completely retained. The height of BME–Ti_3_C_2_T_*x*_ increased to 2.17 nm owing to excessive BME adsorption on the surface of Ti_3_C_2_T_*x*_. Further, the oxidation states of the Ti_3_C_2_T_*x*_ nanosheets were studied using XPS. Figure 2b shows high‐resolution XPS spectra of Ti 2p in pristine Ti_3_C_2_T_*x*_, Ti_3_C_2_T_*x*_–28, and BME–Ti_3_C_2_T_*x*_–28. The Ti 2p spectra were fitted to Ti—C, Ti^2+^, Ti^3+^, TiO_2_, and C—TiF_*x*_ peaks. The peak at a≈ 459.00 eV can be attributed to TiO_2_, which indicates the oxidation degree of MXene.^[^ ^29^ ^]^ For both pristine Ti_3_C_2_T_*x*_ and BME–Ti_3_C_2_T_*x*_–28 samples, the small TiO_2_ peaks reflect the low atomic percentage in the Ti 2p region of ≈10.04 and 12.45 atom%, respectively (Table S1, Supporting Information, presents the atomic ratio obtained by the Ti 2p peak fitting). Nevertheless, for the sample stored in pure water for 28 d, the high TiO_2_ peak reflects a considerably higher atomic percentage of ≈58.32 atom% in the Ti 2p region, which reveals the severe oxidation of Ti_3_C_2_T_*x*_ in water without BME (Figure S7, Supporting Information, depicts the C 1*s* and O 1*s* core levels). To further analyze the effectiveness of BME in stabilizing the Ti_3_C_2_T_*x*_ lattice, TEM measurements were conducted to characterize the morphologies of pristine Ti_3_C_2_T_*x*_, Ti_3_C_2_T_*x*_–28, and 0.005 and 0.05 BME–Ti_3_C_2_T_*x*_–28, as shown in Figure 2c and S8, Supporting Information. After oxidation in pure water for 28 d, a large number of aggregated large particles appeared on the surface of the survived Ti_3_C_2_T_*x*_, consistent with the AFM image, which are probably TiO_2_ particles based on the XPS analysis. The Ti_3_C_2_T_*x*_ nanosheet in the BME solution stored for 28 d still maintained its 2D structure, with an intact lattice like that of a pristine Ti_3_C_2_T_*x*_ nanosheet according to the fast Fourier transform pattern (inset of Figure 2c).^[^ ^30^ ^]^ In addition, Raman spectroscopy was used to monitor the oxidation state of Ti_3_C_2_T_*x*_, as shown in Figure S9, Supporting Information. The characterized A_1g_ (Ti, O, C) vibrational mode of Ti_3_C_2_T_*x*_ was detected at ≈200 cm^−1^ in pristine Ti_3_C_2_T_*x*_, Ti_3_C_2_T_*x*_–28, and 0.05 BME–Ti_3_C_2_T_*x*_–28. Another obvious peak at ≈150 cm^−1^ appeared only in the Ti_3_C_2_T_*x*_–28 sample and was attributed to the *E*_1g_ mode of anatase TiO_2_ formed by the oxidation reaction.[^17^, ^31^] Furthermore, the 0.05 BME–Ti_3_C_2_T_*x*_ sample stored as long as 2 m (BME–Ti_3_C_2_T_*x*_–60) was also characterized by the AFM, XPS, TEM, and Raman in Figure S10, Supporting Information. The 60 d sample retains typical features of Ti_3_C_2_T_*x*_ although the oxidation degree is increased compared to 0.05 BME–Ti_3_C_2_T_*x*_–28 (as discussed in the Supporting Information), indicating that Ti_3_C_2_T_*x*_ can survive for 2 m under the protection of BME.

**Figure 3:**
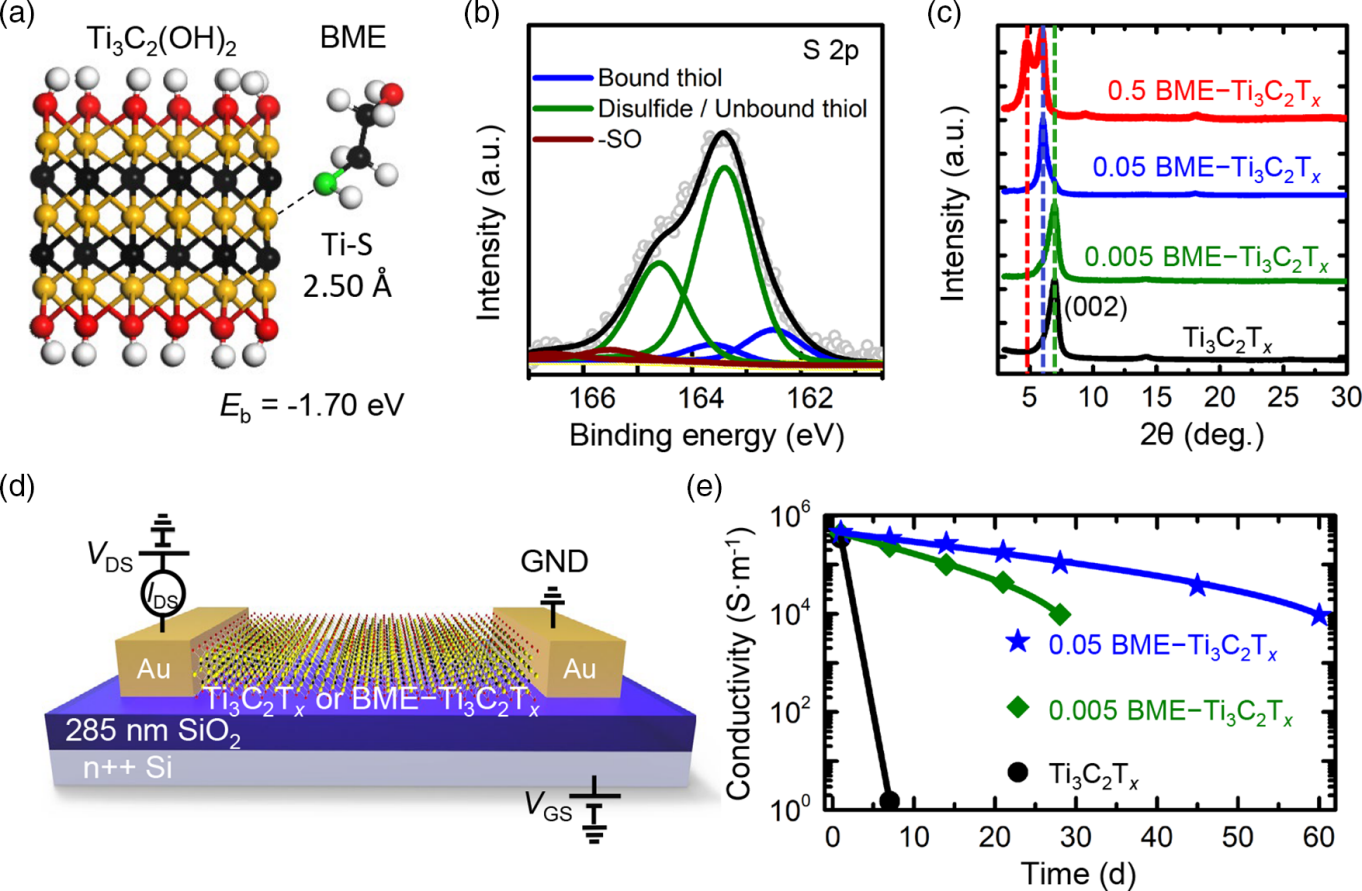
a) Configuration structure of the BME adsorbed to the Ti_3_C_2_(OH)_2_ edge with the corresponding Ti—S bond length and binding energy (*E*_be_). b) S 2p XPS spectra of 0.005 BME–Ti_3_C_2_T_*x*_. c) X‐ray diffraction (XRD) patterns of pristine Ti_3_C_2_T_*x*_ and BME–Ti_3_C_2_T_*x*_ (0.005, 0.05, and 0.5) in a range of diffraction angles from 4° to 30°. d) Schematic of the pristine Ti_3_C_2_T_*x*_/BME–Ti_3_C_2_T_*x*_‐based field‐effect transistor (FET) device. e) Conductivity changes of Ti_3_C_2_T_*x*_ and 0.005 and 0.05 BME–Ti_3_C_2_T_*x*_ flakes on SiO_2_ substrates in humid air over time.

To reveal the mechanism by which BME mitigates the Ti_3_C_2_T_*x*_ degradation, DFT calculations^[^ ^32^ ^]^ were performed to investigate the interaction between —SH groups of BME and Ti_3_C_2_T_*x*_, including the positively charged unpassivated titanium atoms at the edge, the typical single Ti atom defect site and functional groups on the surface of Ti_3_C_2_T_*x*_. **Figure** **3**a presents the optimal structure of BME at the edge of the OH‐terminated Ti_3_C_2_T_*x*_ based on DFT calculations (refer to the DFT calculations in Experimental Section for details). The sulfur atom was aligned between two unsaturated titanium atoms with a Ti—S bond with a bond length of 2.50 Å and binding energy of −1.70 eV. The OH‐terminated MXene plays a major role in the oxidation reaction because the stability of MXene follows OH < —F < =O as the respective groups.^[^ ^33^ ^]^ In addition, most —OH functional groups disappeared in the O 1*s* core level of Ti_3_C_2_T_*x*_–28 (Figure 2b). The three optimized BME adsorption conformations on the surface of OH‐terminated Ti_3_C_2_T_*x*_ are shown in Figure S11, Supporting Information. The —SH groups of BME tend to interact with the Ti_3_C_2_(OH)_2_ surface through the S···H hydrogen bond at a binding energy of −0.68 eV. In addition, as the number of =O groups in the proposed MXene was large according to the XPS O 1*s* peak, the interaction between the BME and Ti_3_C_2_O_2_ has been presented and discussed in Figure S12, Supporting Information. BME is preferentially adsorbed to the edge of MXene rather than to the surface for either Ti_3_C_2_(OH)_2_ or Ti_3_C_2_O_2_. Thus, it can effectively mitigate the oxidation of MXene because the oxidation reaction starts at the edge rather than at the perfect surface. The other oxidation reaction site is the defect on the surface of MXene. Therefore, we constructed a typical single Ti atom defect in Ti_3_C_2_O_2_ and calculated the interaction between the BME and the defect site.^[^ ^34^ ^]^ As shown in Figure S13, Supporting Information, both —SH and —OH of BME have a strong binding energy, exceeding 1 eV on the defect of MXene. It is stronger than that in the case of a perfect surface and BME can still strongly bind to the edge of the defective MXene. Furthermore, the Ti—S bond was experimentally proved. As shown in Figure 3b, the S 2p peaks in the XPS spectra of the aged 0.005 BME–Ti_3_C_2_T_*x*_ sample provide further evidence of the interaction between —SH and Ti_3_C_2_T_*x*_. The peak at 162.5 eV and its shoulder at 163.6 eV is a vital signal, which represents the bound S atom with the bare Ti atom (i.e., forming Ti—S bonds) at the edge or defect of Ti_3_C_2_T_*x*_.^[^ ^35^ ^]^ The peaks at 163.4 eV (164.6 eV for S 2p_1/2_) were assigned to the disulfide bonds originated by the oxidation of thiol groups and unbound thiol groups.^[^ ^36^ ^]^ Figure S14a–c, Supporting Information, shows the binding energies of the S 2p core levels in the XPS spectra of the pristine Ti_3_C_2_T_*x*_, 0.05 and 0.5 BME–Ti_3_C_2_T_*x*_, respectively. The XRD patterns shown in Figure 3c reveal the (002) peaks of pristine Ti_3_C_2_T_*x*_ and BME–Ti_3_C_2_T_*x*_ in the range of 4°–30°, corresponding to the *d* spacing between the Ti_3_C_2_T_*x*_ layers.^[^ ^37^ ^]^ As the amount of BME increases, the interlayer spacing of Ti_3_C_2_T_*x*_ increases from 1.27 nm for the pristine Ti_3_C_2_T_*x*_ to 1.28, 1.46, and 1.48 nm for the 0.005, 0.05, and 0.5 BME–Ti_3_C_2_T_*x*_, respectively (Table S2, Supporting Information, summarizes the layer distances). Notably, the layer spacings of the pristine Ti_3_C_2_T_*x*_ and 0.005 BME–Ti_3_C_2_T_*x*_ are almost equal, because a small amount of BME is preferentially combined with the edge of Ti_3_C_2_T_*x*_. The 0.2 nm increase in layer spacing for 0.05 BME–Ti_3_C_2_T_*x*_ compared to pristine Ti_3_C_2_T_*x*_ may result from more BME adsorbed on the defective (preferentially) and perfect Ti_3_C_2_T_*x*_ surface, but some BME molecules may be removed during the drying process of prepared XRD samples. The (002) peak of 0.5 BME–Ti_3_C_2_T_*x*_ split into two peaks at 5.98° and 4.72° by the uneven intercalation of excessive BME. Thermogravimetric analysis (TGA) and differential scanning calorimetry (DSC) were conducted to analyze the thermal stability of the BME–Ti_3_C_2_T_*x*_ and, in particular, the desorption process of BME. Figure S15a, Supporting Information, shows an additional weight loss of ≈3% occurred at 160–210 °C in BME–Ti_3_C_2_T_*x*_ as compared with pristine Ti_3_C_2_T_*x*_, which was related to the evaporation and desorption of BME. The DSC peaks (Figure S15b, Supporting Information) of BME–Ti_3_C_2_T_*x*_ at 102.7, 160.4, 176.7, and 209.5 °C could be attributed to the evaporation of H_2_O and free BME, and to the desorption of BME on the surface and at the edge of Ti_3_C_2_T_*x*_, respectively. Electron paramagnetic resonance (EPR) was used to study the single electrons in pristine Ti_3_C_2_T_*x*_ and BME–Ti_3_C_2_T_*x*_, as shown in Figure S16, Supporting Information. Consistent with the previous study,^[^ ^38^ ^]^ both pristine Ti_3_C_2_T_*x*_ and BME–Ti_3_C_2_T_*x*_ showed negligible EPR signals, indicating that the strong binding energy between BME and Ti_3_C_2_T_*x*_ edges originated from the electron donation of sulfhydryl groups rather than any possible radical reaction.

To quantitatively study the humid air stability and electrical properties of single‐layer pristine Ti_3_C_2_T_*x*_ and BME–Ti_3_C_2_T_*x*_, FET devices were fabricated using MXene as a channel. Two Au electrodes were thermally deposited to contact MXene on the Si/SiO_2_ substrate, as shown in Figure 3d. The transfer and output characteristics of the pristine Ti_3_C_2_T_x_‐ and BME–Ti_3_C_2_T_*x*_‐based FETs are presented in Figure S17a,b, Supporting Information, respectively. Consistent with the transfer curve, the output curve exhibits a slight n‐type characteristic, and the ultra‐linear *I*_d_–*V*_d_ plot indicates Ohmic contact between the Au electrodes and (BME−)Ti_3_C_2_T_*x*_; thus, the conductivity can be directly calculated from the current. Figure 3e shows the changes in electrical conductivity of pristine Ti_3_C_2_T_*x*_ and 0.005 and 0.05 BME–Ti_3_C_2_T_*x*_ over time for 28 and 60 d, respectively. Previous studies^[^ ^39^ ^]^ have extensively demonstrated the effect of molecular doping on the Ti_3_C_2_T_*x*_ conductivity, and the current experiments indicate that incorporating BME with Ti_3_C_2_T_*x*_ slightly improves the conductivity from 3534.1 to 4371.7 and 4514.9 S cm^−1^ for 0.005 and 0.05 BME–Ti_3_C_2_T_*x*_, respectively. In addition, Ti_3_C_2_T_*x*_ degenerates into an insulator (0.015 S cm^−1^) after 7 d owing to oxidation, whereas 0.005 and 0.05 BME–Ti_3_C_2_T_*x*_ retain excellent conductivities (96.5 and 1082.8 S cm^−1^) even after 28 d. Furthermore, 0.05 BME–Ti_3_C_2_T_*x*_ retains a strong conductivity of 92.6 S cm^−1^ after 60 d, confirming the remarkable stability of BME–Ti_3_C_2_T_*x*_ in humid air. The degradation of BME–Ti_3_C_2_T_*x*_ in air also followed the exponential decay, and the accelerated degradation rate over time may be due to the gradual desorption of BME. Those results demonstrate that BME–Ti_3_C_2_T_*x*_ can be easily fabricated onto 2D flakes on SiO_2_ substrates, and the potential of BME to continuously protect Ti_3_C_2_T_*x*_ in subsequent applications.

**Figure 4:**
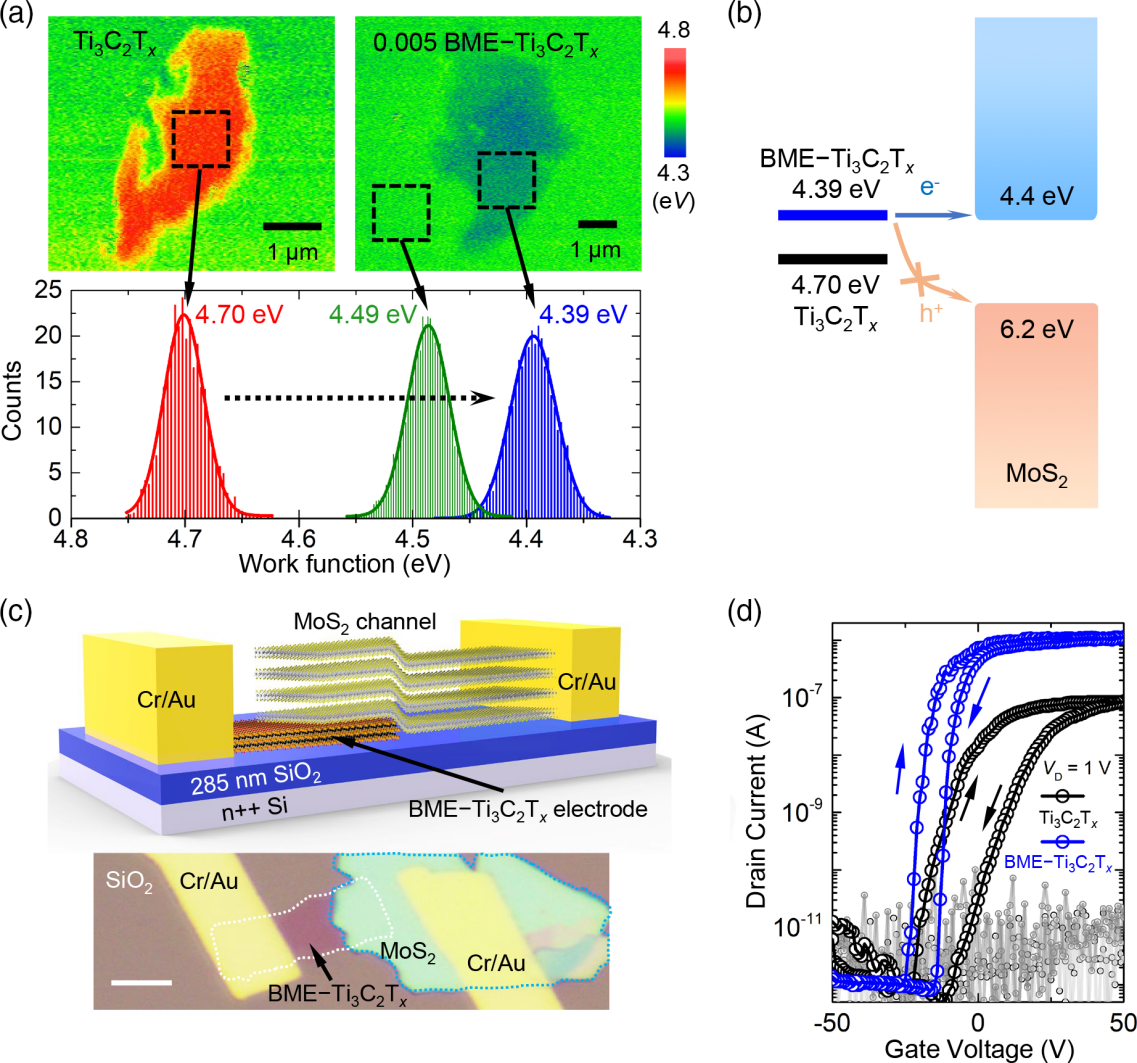
a) Work function maps obtained via Kelvin probe force microscopy (KPFM) (top) and areal work function distributions and Gaussian fitting (bottom) of the pristine Ti_3_C_2_T_*x*_ and 0.005 BME–Ti_3_C_2_T_*x*_. b) Band structures of pristine Ti_3_C_2_T_*x*_, BME–Ti_3_C_2_T_*x*_, and multilayer MoS_2_. c) Schematic (top) and optical image (bottom, scale bar: 5 μm) of the MoS_2_ and BME–MXene FET device. d) Typical transfer characteristics of MoS_2_ and pristine Ti_3_C_2_T_*x*_ (black)/BME–Ti_3_C_2_T_*x*_ (blue) FET devices. The light and dark gray lines indicate the gate current.

**Figure** **4**a presents work function maps of the pristine Ti_3_C_2_T_*x*_ and 0.005 BME–Ti_3_C_2_T_*x*_ sheets on the Si substrates obtained by Kelvin probe force microscopy (KPFM) measurements.^[^ ^40^ ^]^ An evident color contrast is observed, from red (pristine Ti_3_C_2_T_*x*_) to baby blue (BME–Ti_3_C_2_T_*x*_), indicating the successful tuning of the work function. The bottom panel of Figure 4a shows work function distributions in the selected area of the pristine Ti_3_C_2_T_*x*_ (red), Si substrate (green), and 0.005 BME–Ti_3_C_2_T_*x*_ (blue), and their average work functions determined by Gaussian fitting are 4.70, 4.49, and 4.39 eV, respectively. The change of work function can be attributed to the electron‐donating effect of BME, which changes the surface dipole moment of Ti_3_C_2_T_*x*_ and the doping of the conductive layer. Figure S18a,b, Supporting Information, presents a work function map of 0.05 BME–Ti_3_C_2_T_*x*_ and statistical work function (4.40 eV), which indicates that the doping of Ti_3_C_2_T_*x*_ is saturated by 0.005 m BME. Owing to the 0.31 eV amplitude modulation, the work function of BME–Ti_3_C_2_T_*x*_ is very close to the conduction band (CB) or lowest unoccupied molecular orbital of the n‐type 2H‐phase MoS_2_. Figure 4b presents a schematic band diagram of the pristine Ti_3_C_2_T_*x*_, BME–Ti_3_C_2_T_*x*_, and multi‐layered MoS_2_. The work function of BME–Ti_3_C_2_T_*x*_ matches the CB of MoS_2_ (≈4.4 eV),^[^ ^41^ ^]^ which facilitates the barrier‐free injection of electrons. In contrast, when electrons are injected from the pristine Ti_3_C_2_T_*x*_ into MoS_2_, an energy barrier must be overcome. In the case of hole transport, BME–Ti_3_C_2_T_*x*_ encounters larger obstacles than pristine Ti_3_C_2_T_*x*_ to inject holes into the valence band (VB, ≈6.2 eV) of MoS_2_.^[^ ^42^ ^]^ To verify this claim, MoS_2_ FET devices were fabricated to compare the qualities of pristine Ti_3_C_2_T_*x*_ and BME–Ti_3_C_2_T_*x*_ as source electrodes, as shown in Figure 4c. The fabrication is described in Experimental Section. The bottom panel of Figure 4c shows an OM image of a typical MoS_2_ FET device with a BME–Ti_3_C_2_T_*x*_ source electrode. Figure 4d compares the transfer curves of the as‐fabricated FETs using Ti_3_C_2_T_*x*_ (black curve) and BME–Ti_3_C_2_T_*x*_ (blue curve) electrodes. The BME–Ti_3_C_2_T_*x*_ exhibits a high on/off ratio of 10^6^ with an ON current 10 times higher than that of pristine Ti_3_C_2_T_*x*_. Figure S20, Supporting Information, depicts the output curves at various gate voltages. A nonlinear *I*_d_–*V*_d_ for a low *V*_d_ is observed in the pristine Ti_3_C_2_T_*x*_ based FETs, indicating a typical Schottky contact, while a linear *I*_d_–*V*_d_ supports Ohmic contact between BME–Ti_3_C_2_T_*x*_ and MoS_2_. In addition, Figure S21, Supporting Information, shows that the performance of the FETs with the monolayer BME–Ti_3_C_2_T_*x*_ electrodes is comparable to that of conventional thick Cr/Au (5/50 nm) electrodes, demonstrating the potential of BME–Ti_3_C_2_T_*x*_ as a 2D electrode in future electronics.

## 3 Conclusion

We have demonstrated that the BME treatment could suppress the oxidation of Ti_3_C_2_T_*x*_ MXene, in the colloidal dispersion (2 m), under humid air conditions (2 m), or even in harsh 100 °C water (12 h). The absorption mechanism between BME and Ti_3_C_2_T_*x*_ was investigated by comprehensive DFT calculations and experimental characterizations, revealing BME has strong interaction with both the edge (through Ti—S bond, binding energy −1.70 eV) and defect (by hydrogen bond, binding energy −1.05 eV) of Ti_3_C_2_T_*x*_, which significantly suppresses the degradation of Ti_3_C_2_T_*x*_. The work function of Ti_3_C_2_T_*x*_ was successfully tuned from 4.70 to 4.39 eV by the BME treatment because of the electron‐donating effect of sulfhydryl groups. BME–Ti_3_C_2_T_*x*_ showed excellent electrical properties and much better humid air stability than Ti_3_C_2_T_*x*_ in as‐fabricated FET devices, and served as a better source electrode material than pristine Ti_3_C_2_T_*x*_ in n‐type MoS_2_ FETs because of the band alignment effect. The described methodology can largely contribute to the long‐term storage and service life of Ti_3_C_2_T_*x*_ and simultaneously cover every aspect (in comparison to recently published antioxidant methods, see Table S3, Supporting Information), thus promoting the practical application of MXene. In addition, the elucidation of the strong interaction between sulfhydryl groups with MXene and the improved stability of MXene at high temperatures are expected to inspire related studies and further expand the research field of MXene.

## 4 Experimental Section

4.1

4.1.1

##### Synthesis of Ti_3_C_2_T_x_

To remove the Al layer in Ti_3_AlC_2_ by chemical etching, 1 g of Ti_3_AlC_2_ was slowly added to the etchant (precooled to below 5 °C) containing 1.6 g of LiF (Sigma Aldrich), 5 mL of deionized H_2_O, and 15 mL of 12 m HCl (hydrochloric acid, Sigma Aldrich) in a 100 mL Teflon beaker. The mixture with a Teflon stirring bar was treated by N_2_ blowing and then sealed prior to being placed in an ice bath and stirred at 500 rpm for 1 h. Subsequently, the reaction was continued at 25 °C for 36 h. The product was then washed with centrifugation (5–6 cycles, at 3500 rpm) and manual shaking until the pH of the supernatant became neutral. The mixture was then centrifuged at 3500 rpm for 1 h, and the resulting colloid and swollen upper black precipitate were collected for follow‐up experiments. The concentration of the as‐prepared Ti_3_C_2_T_*x*_ dispersion was determined to be approximately 13 mg mL^−1^ by the freeze‐drying technology.^[^ ^43^ ^]^

##### Protection of Ti_3_C_2_T_x_ by BME

Typically, in a 10 mL vial, 2.5 mL of Ti_3_C_2_T_*x*_ obtained as described earlier was diluted with 7.5 mL of deionized water or BME solution, which yielded final BME concentrations in the 10 mL Ti_3_C_2_T_*x*_ colloid of 0.005, 0.05, and 0.5 mol L^−1^. The ratios of BME and Ti_3_C_2_T_*x*_ were ≈0.12:1 (0.005 BME–Ti_3_C_2_T_*x*_), 1.2:1 (0.05 BME–Ti_3_C_2_T_*x*_), and 12:1 (0.5 BME–Ti_3_C_2_T_*x*_), respectively. Thereafter, manual shaking was performed for ≈10 min to thoroughly mix Ti_3_C_2_T_*x*_ and BME, followed by sonication in an ice water bath for 3 min to separate the oxidized Ti_3_C_2_T_*x*_ maximally. The floating impurities on the dispersion surface were carefully removed and, finally, the vials were capped and stored in a dark place.

##### Device Fabrication

The highly doped n‐type Si and thermally grown 285 nm thick SiO_2_ served as the bottom gate and dielectric layer, respectively. The ten times diluted pristine Ti_3_C_2_T_*x*_ or BME–Ti_3_C_2_T_*x*_ dispersion was spin‐coated on the O_2_ plasma‐treated substrate. An MXene flake with a regular shape was selected through OM and determined as a monolayer through AFM. For the fabrication of MXene‐based FET devices, poly(methyl methacrylate) (PMMA) was coated onto the pristine Ti_3_C_2_T_*x*_ and BME–Ti_3_C_2_T_*x*_ samples, followed by electron‐beam lithography (EBL) to pattern the source–drain electrodes. Subsequently, Cr (5 nm)/Au (50 nm) was deposited through e‐beam evaporation. For the fabrication of MoS_2_‐MXene FETs, multilayer MoS_2_ flakes were mechanically exfoliated from bulk MoS_2_ crystals. High‐quality MoS_2_ flakes with suitable sizes (thicknesses ≈ 20 nm) were attached to a polydimethylsiloxane (PDMS) stamp on a glass slide and then transferred onto the selected MXene (using OM and AFM) surface in a nitrogen‐filled glove box. After stacking, the resulting pristine Ti_3_C_2_T_*x*_/BME–Ti_3_C_2_T_*x*_‐MoS_2_ samples were immediately spin‐coated with PMMA solution and patterned with EBL to control the channel length as ≈2 μm. Finally, 5/50 nm thick Cr/Au electrodes were deposited as the source and drain electrodes at a low pressure <10^−6^ Torr at a speed of 1 A s^−1^. Subsequently, a metal lift‐off process was performed. The electrical properties of the devices were measured using a Keithley 4200 parameter analyzer at a pressure of 10^−4^ Torr.

##### Sample Characterization

The surface morphologies of the pristine Ti_3_C_2_T_*x*_ and BME–Ti_3_C_2_T_*x*_ flakes were characterized by OM, AFM, and TEM. The thicknesses of the MXene and BME–spin‐coated‐MXene flakes were measured using AFM (Park System Corp.). The work function was measured using the KPFM method in an AFM–Raman measurement system equipped with a Kelvin probe (NTEGRA Spectra, NT‐MDT 830). The contact potential difference (Δ*V*_CPD_) was calculated based on work function measurements collected from the KPFM tip and the sample according to the Equation eΔ*V*_CPD_ = Φ_tip_ − Φ_sample_, where Φ_tip_ is the work function of the tip, Φ_sample_ is the work function of the sample, and e is the electric charge. Φ_tip_ was calibrated at 4.75 eV using the KPFM measurements collected from highly ordered pyrolytic graphite. Optical images were obtained using an OM (Olympus BX51). TEM images were acquired by low‐resolution TEM with an incident electron beam energy of 80 keV (TEM 2100 F, JEOL). The chemical compositions were probed using XPS (ESCA2000, VG Microtech Inc.) in an ultrahigh vacuum with an Mg K_α_ X‐ray source. The absorption properties of the pristine Ti_3_C_2_T_*x*_ and BME–Ti_3_C_2_T_*x*_ dispersion were observed using UV–vis–NIR spectroscopy with injection from a deuterium and tungsten lamp (Shimadzu UV–3600). Raman spectra were acquired using a Raman microscopy system (Kaiser Optical Systems model RXN) fitted with a 532 nm laser. The thermal stability was explored by using a DSC–TGA thermal analyzer (SDT650) in the presence of nitrogen gas. XRD patterns were acquired using Cu KR radiation at a wavelength of 0.1541 nm. EPR signals were obtained using X‐band CW‐EPR at RT with a microwave power of 1 mW, microwave frequency of 9.64 GHz, modulation frequency of 100 kHz, and modulation amplitude of 10 G.

##### DFT Calculation Methods

To probe the interactions between BME and Ti_3_C_2_T_*x*_ in the BME–Ti_3_C_2_T_*x*_ hybrid system, DFT calculations were performed using the DMol^3^^[^ ^44^ ^]^ code. The generalized gradient approximation with the Perdew−Burke−Ernzerhof (PBE) functional and DFT semi‐core pseudo‐potential double numerical atomic basis set plus polarization were employed.^[^ ^45^ ^]^ Owing to the poor description of the weak van der Waals interactions of the popular PBE functional,^[^ ^46^ ^]^ an empirical dispersion‐corrected DFT approach proposed by Grimme was used.^[^ ^47^ ^]^ The optimal geometric convergence criteria of energy, force, and displacement were 1.0 × 10^−5^ Ha, 0.002 Ha Å^−1^, and 0.005 Å, respectively. The *k*‐point grid was set to be 1 × 3 × 1 for structural optimization. The vacuum thickness was set to be more than 20 Å to avoid interactions between neighboring layers. A supercell consisting of 3 × 2 Ti_3_C_2_O_2_ nanoribbons was selected to adsorb BME on the edge and surface for the simulation. A vacuum gap larger than 20 Å was added to the other two nonperiodic directions (*y‐* and *z*‐axes). To quantitatively describe the interaction strength between BME and Ti_3_C_2_T_*x*_, the binding energy was defined as *E*_b_ = *E*_total_−*E*_BME_−*E*_MXene_, where *E*_total_, *E*_BME_, and *E*_MXene_ denote the energies of BME–Ti_3_C_2_T_*x*_, BME, and pristine Ti_3_C_2_T_*x*_ flake, respectively.

## Conflict of Interest

The authors declare no conflict of interest.

## Supporting information

Supplementary Material

## Data Availability

The data that support the findings of this study are available from the corresponding author upon reasonable request.
