# Supplementary material for: β‐Mercaptoethanol‐Enabled Long‐Term Stability and Work Function Tuning of MXene
Source: Small Sci. 2022 Oct 2;2(11):2200057. doi: 10.1002/smsc.202200057 (PMC11936050; doi:10.1002/smsc.202200057)
Supplement: Supplementary file 1 — Supplementary Material [file SMSC-2-2200057-s001.pdf]

Supporting Information

β-Mercaptoethanol-Enabled Long-Term Stability and Work Function Tuning of MXene

*Hongyue Jing[1 †], Benzheng Lyu[2 †], Yingqi Tang[3], Sungpyo Baek[1], Jin-Hong Park[1], Byoung Hun Lee[4], Jin Yong Lee[3], and Sungjoo Lee[1,5]**

[1]*SKKU Advanced Institute of Nanotechnology (SAINT), Sungkyunkwan University, Suwon 440-746, Korea*

[2]*Department of Electrical and Electronic Engineering, The University of Hong Kong, Hong Kong, China*

[3]*Department of Chemistry, Sungkyunkwan University, Suwon 16419, Republic of Korea*

[4]*Department of Electrical Engineering, Pohang University of Science and Technology, Pohang 37673, Korea*

[5]*Department of Nano Engineering, Sungkyunkwan University, Suwon 440-746, Korea*

Without protection measures, $Ti_3C_2T_x$ MXene flakes easily oxidize into TiO_2 and carbon. The oxidation initially occurs at the edge part and surface defects, where Ti dangling bonds exist. Figure S1 shows typical oxidation images of $Ti_3C_2T_x$ flakes obtained by AFM and low-magnification TEM.

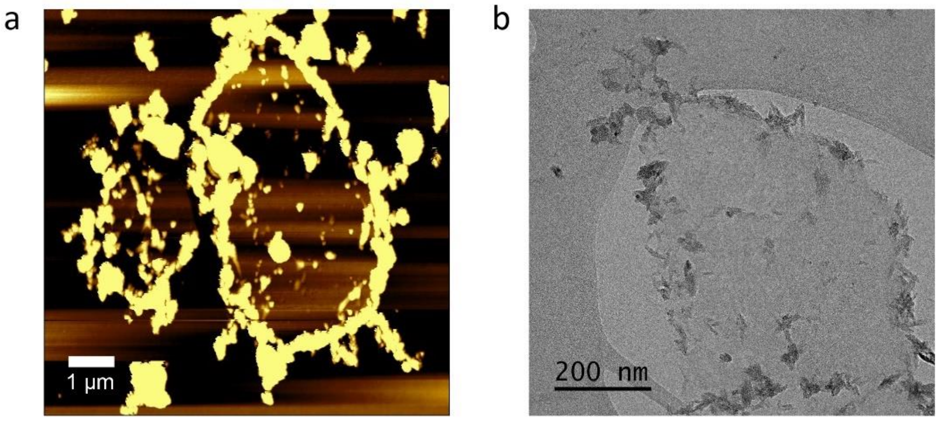

Figure S1. (a) AFM and (b) low-magnification TEM images of oxidized $Ti_3C_2T_x$ flakes.

Figure S2 shows a schematic of the liquid exfoliation of $Ti_3C_2T_x$ flakes using HCl/LiF, where HF acts as the etching reagent and Li^+ ions play a role of delamination.

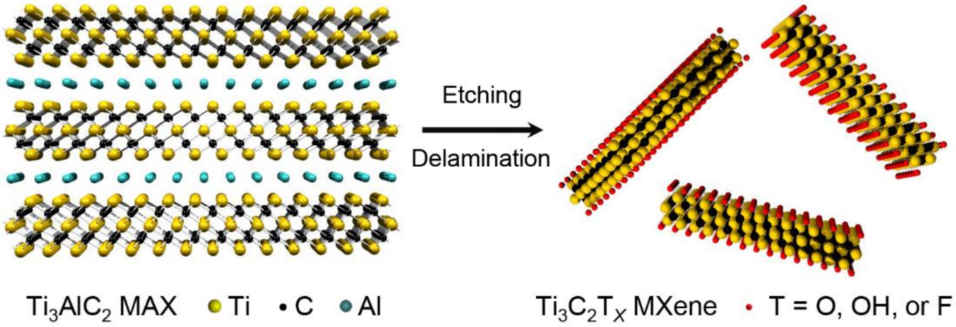

Figure S2. Schematic of the synthesis of $Ti_3C_2T_x$ nanosheets.

Figure S3 shows the UV−Vis−NIR absorption curves of the diluted 0, 0.005, 0.05, and 0.5 BME−$Ti_3C_2T_x$ aged for 28 d at room temperature.

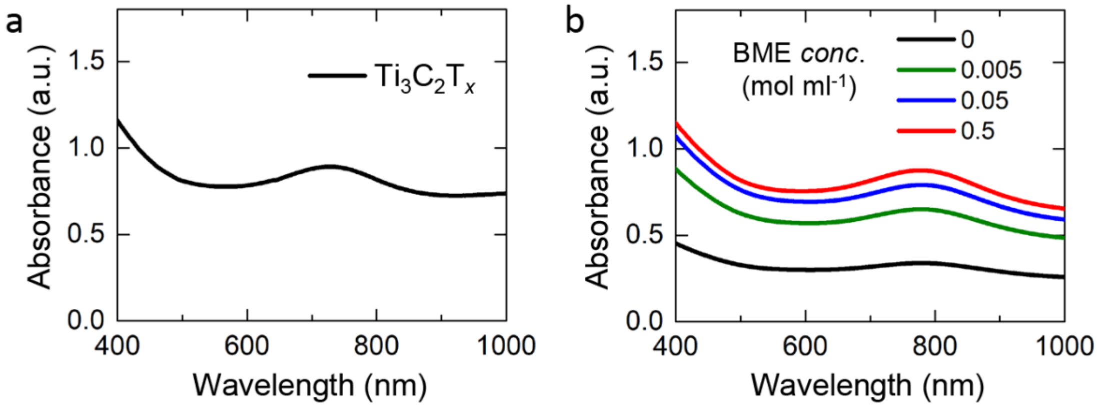

Figure S3. UV−Vis−NIR spectra of the freshly synthesized pristine $Ti_3C_2T_x$ (a), and 28 d aged BME−$Ti_3C_2T_x$ with 0, 0.005, 0.05, and 0.5 M BME at room temperature.

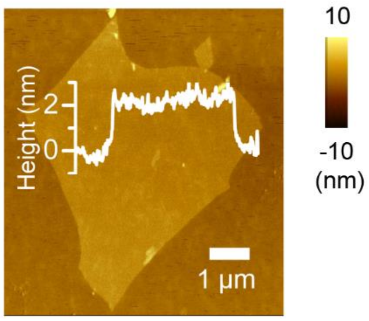

Figure S4. AFM image and extracted height profile of a single-layer 0.05 BME−$Ti_3C_2T_x$ flake in humid air for 4 m.

Figure S5 shows the UV−Vis−NIR absorption curves of the 0, 0.005, 0.05, and 0.5 BME−$Ti_3C_2T_x$ at 100 °C as a function of time. The peak at approximately 750 nm is the typical UV−Vis−NIR absorption peak of $Ti_3C_2T_x$. Its intensity is related to concentration of unoxidized $Ti_3C_2T_x$. Without the protection of BME, $Ti_3C_2T_x$ nanosheets were almost completely decomposed within 3 h. With the addition of BME, $Ti_3C_2T_x$ could still be detected after 12 h, and the retention ratio of $Ti_3C_2T_x$ reached 60.5% when the BME concentration is 0.5 M.

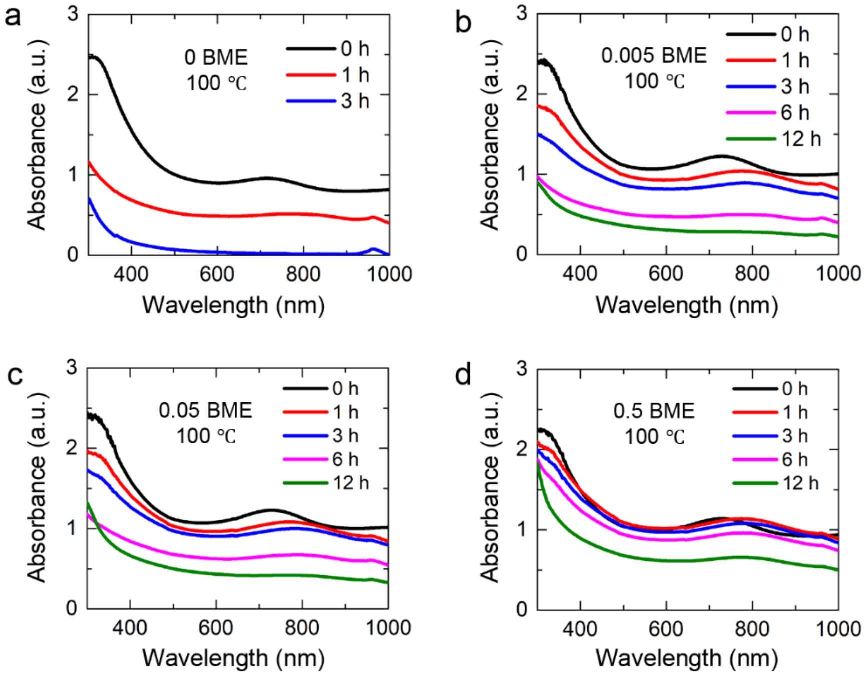

Figure S5. UV−Vis−NIR absorption curves of (a) 0, (b) 0.005, (c) 0.05, and (d) 0.5 BME−$Ti_3C_2T_x$ at 100 °C as a function of time.

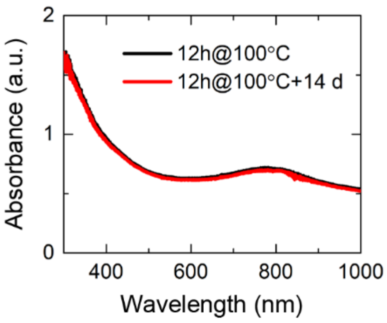

Figure S6. UV−vis−NIR absorption curves of 0.5 BME−$Ti_3C_2T_x$ at 100 °C for 12 h (black curve), and at 100 °C for 12 h and subsequent room temperature for 14 d (red curve).

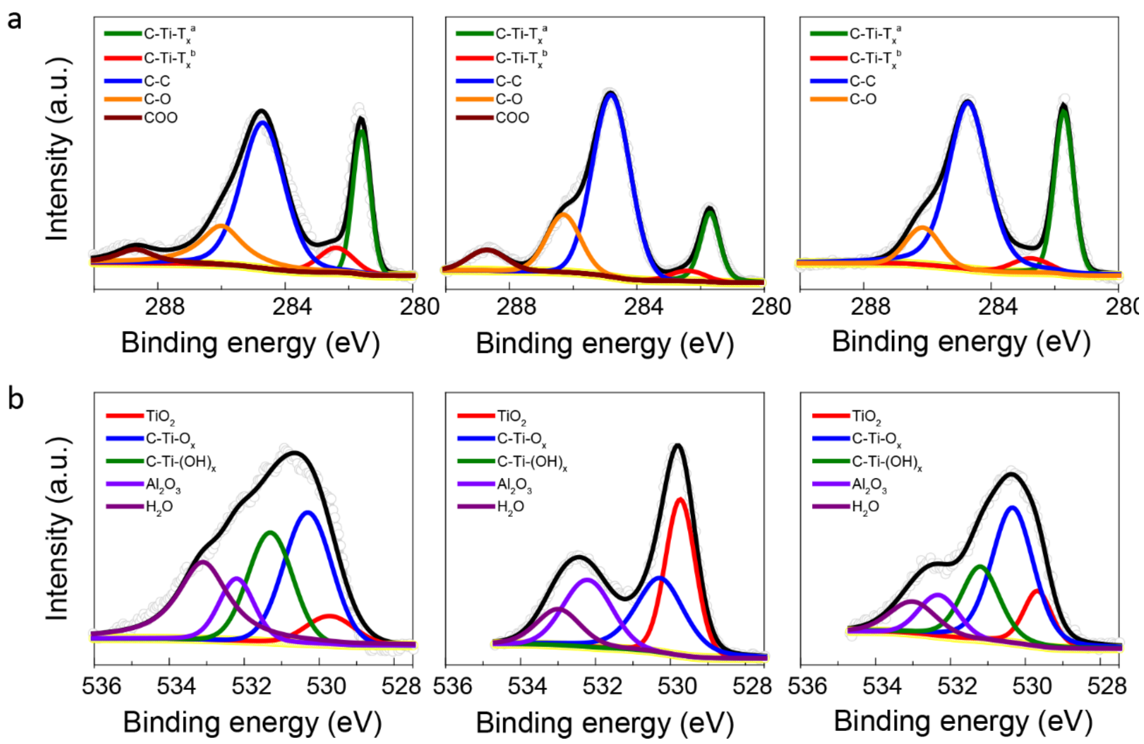

Figure S7. (a) C 1*s* and (b) O 1*s* XPS spectra of pristine $Ti_3C_2T_x$ (left panel), $Ti_3C_2T_x$−28 (middle panel), and 0.05 BME−$Ti_3C_2T_x$−28 (right panel).

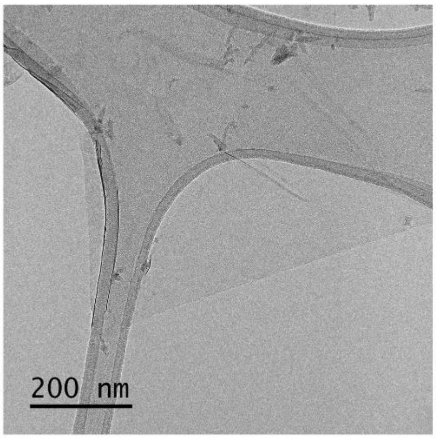

Figure S8. TEM image of 0.005 BME−$Ti_3C_2T_x$−28.

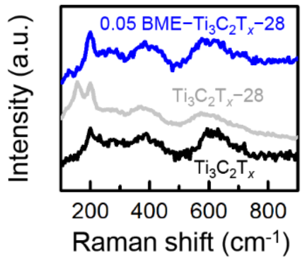

Figure S9. Raman spectra of pristine $Ti_3C_2T_x$, $Ti_3C_2T_x$−28, and 0.05 BME−$Ti_3C_2T_x$−28.

Figure S10a shows an AFM image and corresponding extracted height profile of a 0.05 BME−$Ti_3C_2T_x$–60 nanosheet. The height indicates that the BME−$Ti_3C_2T_x$–60 flake was monolayer, and the AFM image clearly shows that its 2D structure was well preserved. Although slight oxidation (including large particles at the edge, a pinhole of ~1.5 nm in depth, and a bump of ~2 nm in height) were observed, the degree of oxidation of 0.05 BME−$Ti_3C_2T_x$–60 was still considerably less than that of pure $Ti_3C_2T_x$ after 1 m of storage, as shown in Figure 2a (middle panel). Figure S10b shows the XPS spectra of Ti 2p for 0.05 BME−$Ti_3C_2T_x$–60. The TiO_2 peaks at approximately 459.00 eV demonstrated the oxidation state of 0.05 BME−$Ti_3C_2T_x$–60. Compared with the TiO_2 peaks of $Ti_3C_2T_x$−28, the TiO_2 peaks indicated a slight degree of oxidation. TEM micrographs were also obtained to analyze the degree of oxidation of 0.05 BME−$Ti_3C_2T_x$–60 (Figure S10c). The particles at the edge of the TEM image also indicated slight oxidation of BME−$Ti_3C_2T_x$ after 2 m of storage. In addition, Raman spectroscopy was used to monitor the oxidation state of the 60 d BME−$Ti_3C_2T_x$ (Figure S10d). The small peak at approximately 150 cm^{-1} was due to the E_{1g} mode of TiO_2, which was consistent with the AFM, XPS, and TEM results. Overall, MXene can survive for as long as 2 m in the presence of BME.

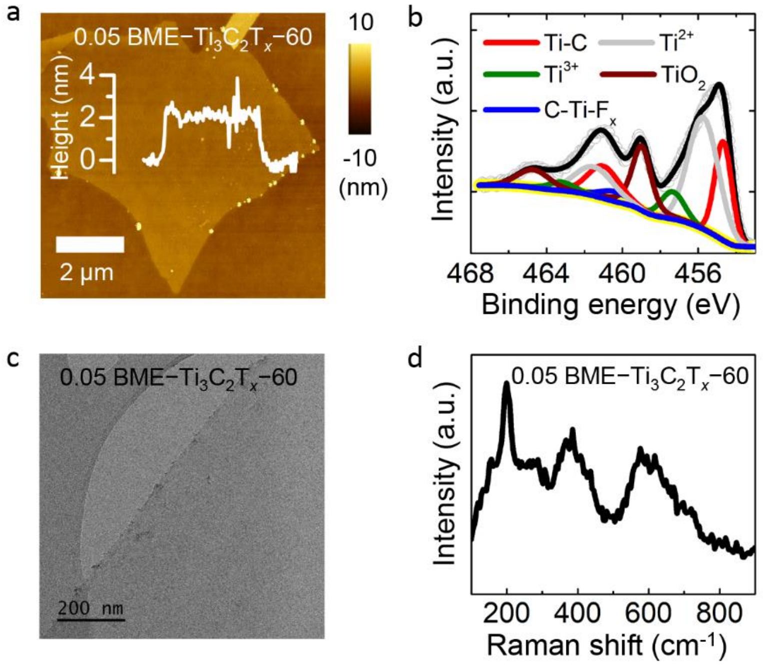

Figure S10. (a) AFM image, (b) XPS spectra, (c) low-resolution TEM micrograph, and (d) Raman spectra of 0.05 BME−$Ti_3C_2T_x$ in solution for 2 m (0.05 BME−$Ti_3C_2T_x$–60).

To build the $Ti_3C_2T_x$ model, fcc-type single -OH or =O functional group was adopted because the terminal groups tend to be on top of the middle layer Ti atoms theoretically and experimentally.[1]

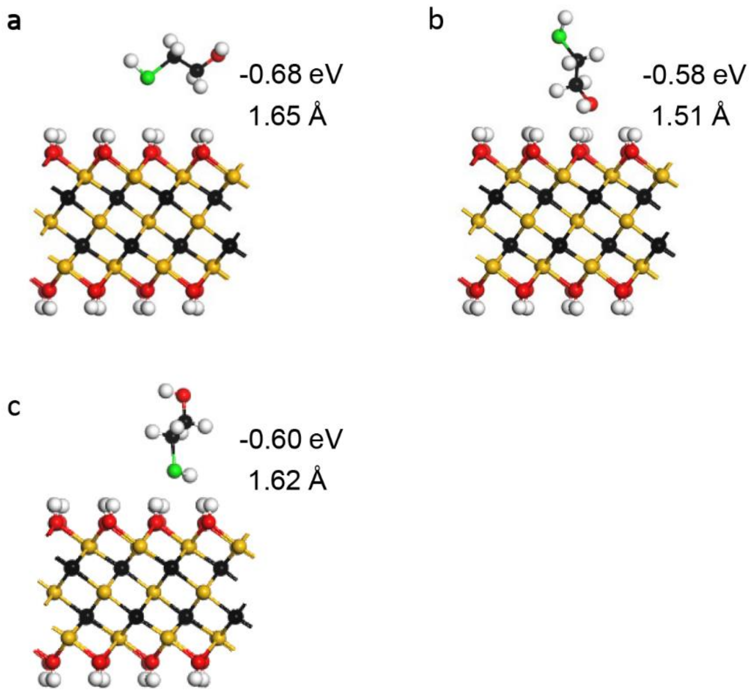

Figure S11. Three configurations and corresponding binding energies and distances of optimized structures of the BME adsorbed to the surface of $Ti_3C_2(OH)_2$ **(**white, red, yellow, black, and olive balls denote the H, O, Ti, C, and S atoms, respectively).

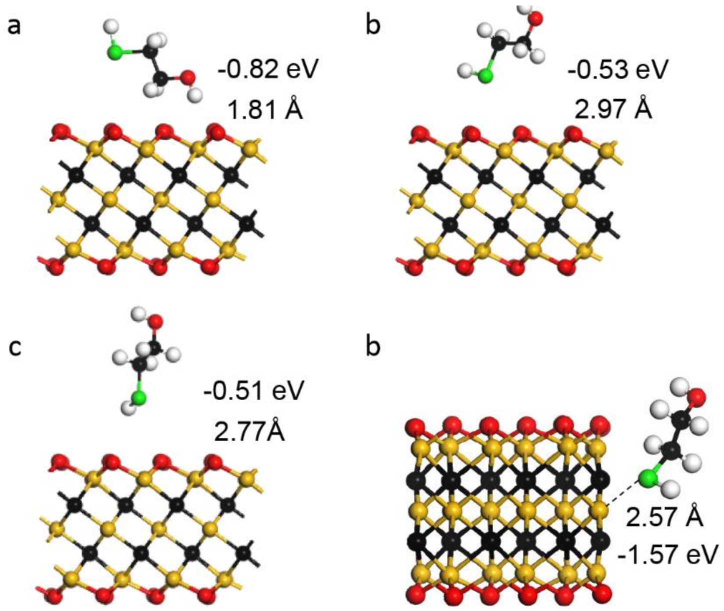

Figure S12. Three configurations (a, b, and c) and their corresponding binding energies, and distances of the optimized structures of the BME adsorbed to the surface of $Ti_3C_2O_2$. (d) Configuration structure of the BME adsorbed to the $Ti_3C_2O_2$ edge with corresponding Ti-S bond length and binding energy (white, red, yellow, black, and olive balls denote the H, O, Ti, C, and S atoms, respectively).

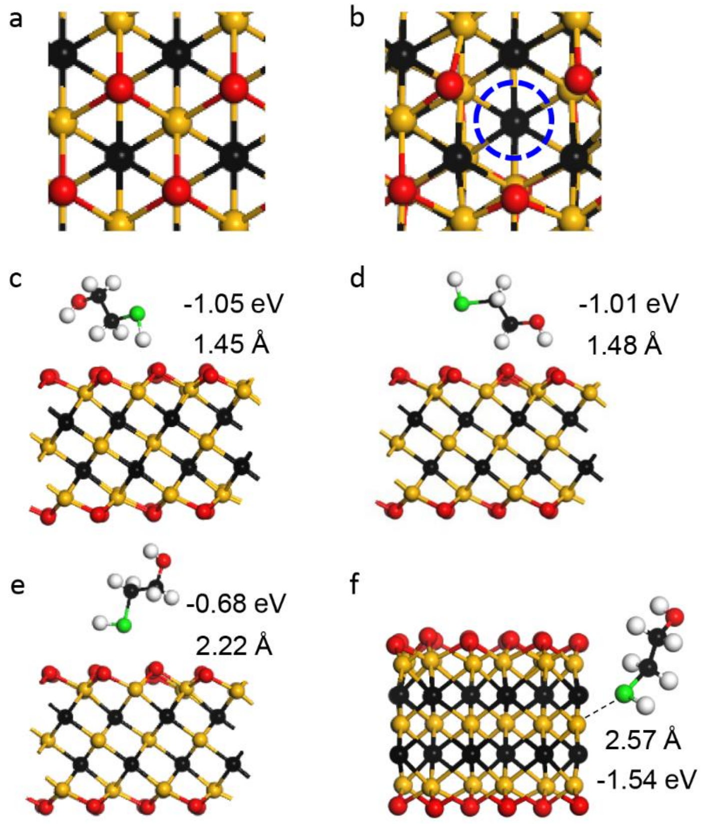

Figure S13. (a) Top view of $Ti_3C_2O_2$ MXene with a perfect surface and (b) single Ti atom defect (indicated by the blue circle); optimal configurations of the BME interacted with the defect site (c, d, and e) and edge (f) of the defect $Ti_3C_2O_2$ (related binding energy, vertical distance from the BME to the $Ti_3C_2O_2$ surface, and Ti-S bond length are given; white, red, yellow, black, and olive balls denote the H, O, Ti, C, and S atoms, respectively).

Previous studies demonstrated that a low-concentration SH retains protons when forming a self-assembled monolayer on a gold surface.[S2] Therefore, −SH in a neutral state was used to prevent the introduction of Coulomb force between Ti^+ and S^-. The S 2*p* peaks in the XPS spectra were thoroughly fitted, assuming that S 2*p* splits into S $2p_{3/2}$ and S $2p_{1/2}$ core levels with a theoretical ratio of 2:1. Notably, no sulfur element was detected in the pristine $Ti_3C_2T_x$ because no sulfur source was introduced during the synthesis of $Ti_3C_2T_x$. The bound thiol was also detected in the XPS spectra of

0.05 BME−$Ti_3C_2T_x$, while it was challenging to fit the bound thiol groups for the 0.5 BME−$Ti_3C_2T_x$ because of the large amount of unbound −SH.

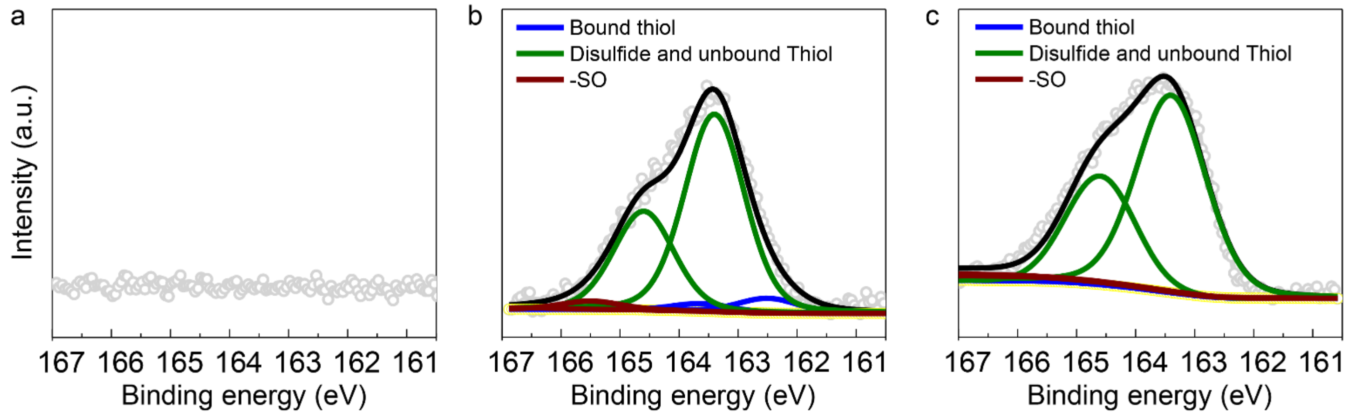

Figure S14. Binding energies of the S 2*p* core levels in the XPS spectra of (a) pristine $Ti_3C_2T_x$, (b) 0.05 BME−$Ti_3C_2T_x$, and (c) 0.5 BME−$Ti_3C_2T_x$.

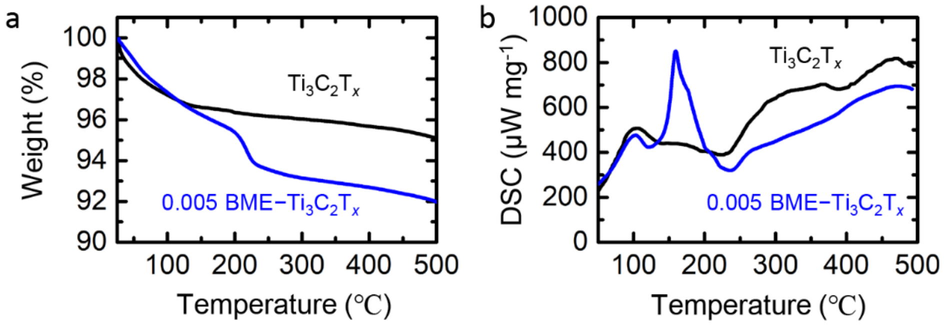

Figure S15. (a) TGA and (b) DSC curves of pristine $Ti_3C_2T_x$ and 0.005 BME−$Ti_3C_2T_x$ in nitrogen atmosphere.

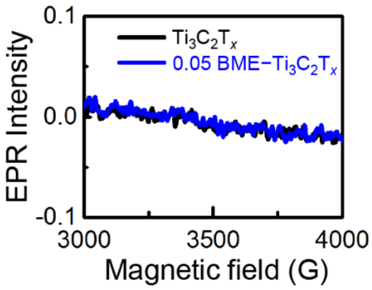

Figure S16. EPR signals of pristine $Ti_3C_2T_x$ and 0.05 BME−$Ti_3C_2T_x$ at room temperature.

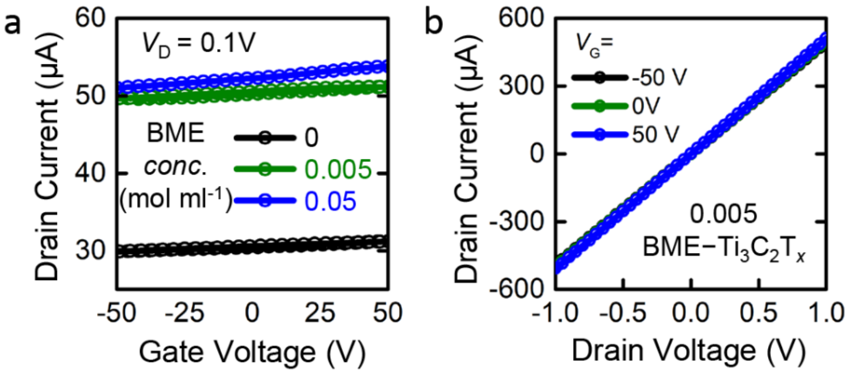

Figure S17. Transfer (a) and output (b) characteristics of the pristine $Ti_3C_2T_x$− and BME−$Ti_3C_2T_x$−based FETs.

The majority carriers in pristine $Ti_3C_2T_x$ are electrons because I_d increases with the V_g sweep in the positive direction. As the concentration of BME increases, the transfer curve of the pristine $Ti_3C_2T_x$−based FET drifted to the high current region. The ultralinear I_d−V_d indicates an Ohmic contact between the Au electrodes and MXene. Thus, the conductivity of MXene can be directly calculated using the current. The conductivities of pristine $Ti_3C_2T_x$ and BME−$Ti_3C_2T_x$ were calculated from the output curves, as $\sigma = LI_d/(V_dWT)$, where T is the thickness of the single-layer pristine $Ti_3C_2T_x$ or BME−$Ti_3C_2T_x$ obtained using the XRD pattern.[S3]

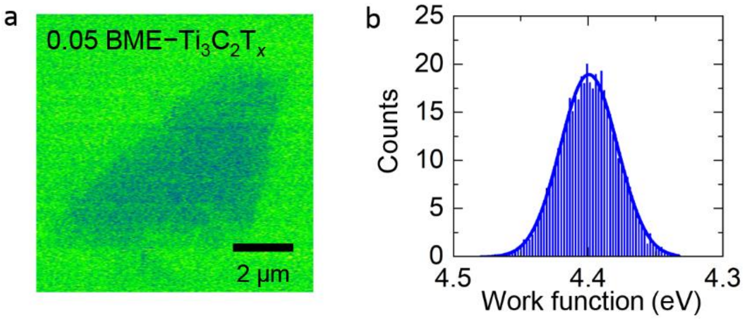

Figure S18. (a) Work function map and (b) statistical work function of 0.05 BME−$Ti_3C_2T_x$.

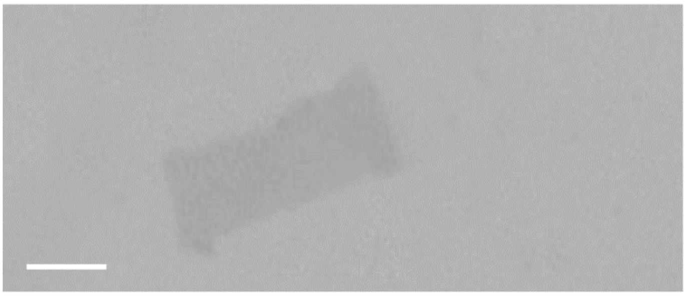

Figure S19. Optical image (scale bar: 5 μm) of the 0.005 BME−$Ti_3C_2T_x$ electrode used in the MoS_2 based FET device.

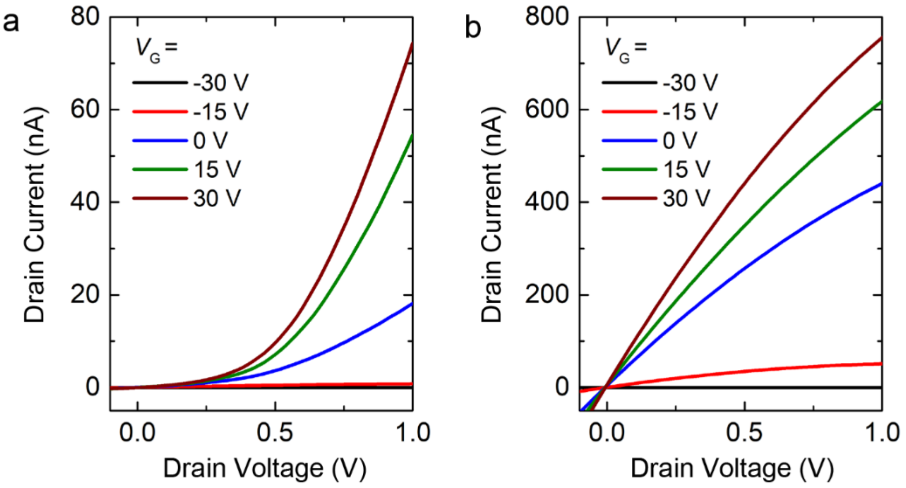

Figure S20. Output characteristics of (a) pristine $Ti_3C_2T_x$-based and (b) BME−$Ti_3C_2T_x$-based MoS_2 FET devices for different gate voltages.

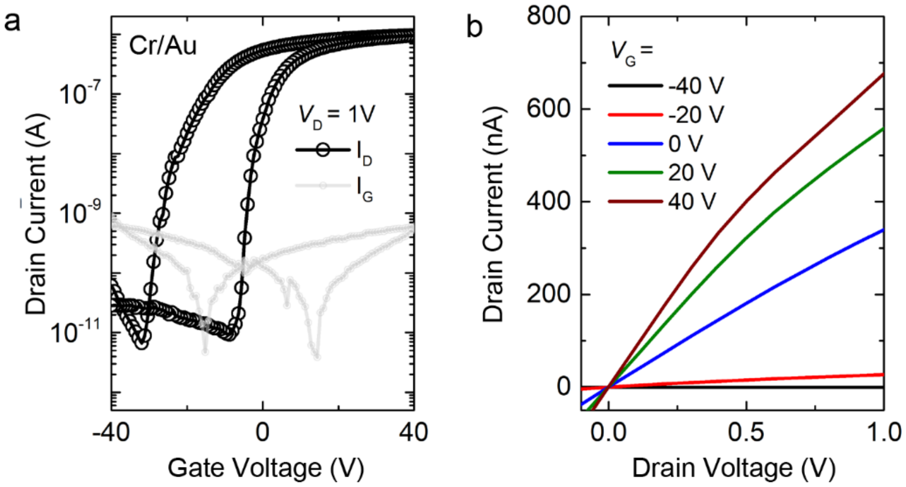

Figure S21. (a) Transfer and (b) output characteristics of Cr/Au-electrode-based MoS_2 FETs.

Table S1. Atomic ratios of Ti with different oxidation states obtained by XPS Ti 2*p* peak fitting.

Ti $2p_{3/2}$	Ti–C	Ti^{2+}	Ti^{3+}	TiO_2	C–Ti–F_x
$Ti_3C_2T_x$	16.8	42.17	12.03	10.04	18.95
$Ti_3C_2T_x$−28	8.91	23.96	6.96	58.32	1.85
BME−$Ti_3C_2T_x$−28	15.78	52.76	13.69	12.45	5.32

Table S2. (002) peak positions and interlayer distances of the pristine $Ti_3C_2T_x$ and 0.005, 0.05, and 0.5 BME−$Ti_3C_2T_x$.

Material	2θ/°	*d* spacing/nm
Pristine $Ti_3C_2T_x$	6.98	1.27
0.005 BME−$Ti_3C_2T_x$	6.92	1.28
0.05 BME−$Ti_3C_2T_x$	6.06	1.46
0.5 BME−$Ti_3C_2T_x$[a]	5.98	1.48
	4.72	1.88

[a)] The (002) peak of 0.5 BME−$Ti_3C_2T_x$ split into two peaks at 5.98° and 4.72° by the uneven intercalation of excessive BME.[S4]

Table S3. Comparison of the recently reported antioxidant methods (O: excellent, Π: lackluster, Δ: unknown or limit).

Method	Mechanism		Antioxidation effect		2D MXene dispersion	Economy (energy and efficiency)	Anti-bacteria
	External factor control	MXene passivation	Edges	Defects			
Degassing[S5]	O	Π	Π[a]	Π	O	O	O
Organic solvent[S6]	O	Π	O	O	Δ[b]	Π	O
Low temperature[S7]	O	Π	O	O	O	Π	O

Defect–free MAX phase[S8]	Π	O	Δ	O	O	O	O
Polyphosphates[S9]	Π	O	O	O	Π	O	O
Sodium L–ascorbate[S10]	O	O	O	O	Δ[c]	O	Π[d]
This study	O	O	O	O	O	O	O

[a)] It is challenging to keep MXene stable with a single degassing process. [b)] Without additives, only a low-concentration small-size MXene can be dispersed in a part of polar organic solvents, while a high-concentration large-size MXene aggregates in most organic solvents. [c)] A high-concentration salt destroys the hydration layer of MXene, thereby causing aggregation. In our experiment, 10 mg mL^{-1} sodium L–ascorbate causes aggregation, while MXene can be stably dispersed in a BME solution (up to 0.5 M (39 mg mL^{-1})) for at least 28 d. Antioxidants will be consumed in some practical applications; thus, the compatibility of MXene and high-concentration antioxidants is important. [d)] Bacteria or mold colonies were judged by visual observation of the surface and inside of the MXene dispersion for 28 d.

References

[1] a) Y. Xie, P. Kent, *Phys. Rev. B* **2013**, *87*, 235441; b) P. O. Persson, J. Rosen, *Curr. Opin. Solid State Mater. Sci.* **2019**, *23*, 100774; c) J. Palisaitis, I. Persson, J. Halim, J. Rosen, P. O. Persson, *Nanoscale* **2018**, *10*, 10850.

[2] M. S. Inkpen, Z. F. Liu, H. Li, L. M. Campos, J. B. Neaton, L. Venkataraman, *Nat. Chem.* **2019**, *11*, 351.

[3] S. Wan, S. Fang, L. Jiang, Q. Cheng, R. H. Baughman, *Adv. Mater.* **2018**, *30*, 1802733.

[4] I. Janigová, F. Lednický, D. J. Mošková, I. Chodák, "Nanocomposites with biodegradable polycaprolactone matrix", presented at *Macromol. Symp.*, 2011.

[5] C. J. Zhang, S. Pinilla, N. McEvoy, C. P. Cullen, B. Anasori, E. Long, S.-H. Park, A. s. Seral-Ascaso, A. Shmeliov, D. Krishnan, *Chem. Mater.* **2017**, *29*, 4848.

[6] K. Maleski, V. N. Mochalin, Y. Gogotsi, *Chem. Mater.* **2017**, *29*, 1632.

[7] Y. Chae, S. J. Kim, S.-Y. Cho, J. Choi, K. Maleski, B.-J. Lee, H.-T. Jung, Y. Gogotsi, Y. Lee, C. W. Ahn, *Nanoscale* **2019**, *11*, 8387.

[8] T. S. Mathis, K. Maleski, A. Goad, A. Sarycheva, M. Anayee, A. C. Foucher, K. Hantanasirisakul, C. E. Shuck, E. A. Stach, Y. Gogotsi, *ACS Nano* **2021**, *15*, 6420.

[9] V. Natu, J. L. Hart, M. Sokol, H. Chiang, M. L. Taheri, M. W. Barsoum, *Angew. Chem. Int. Ed.* **2019**, *131*, 12785.

[10] X. Zhao, A. Vashisth, E. Prehn, W. Sun, S. A. Shah, T. Habib, Y. Chen, Z. Tan, J. L. Lutkenhaus, M. Radovic, *Matter* **2019**, *1*, 513.
